# Extending reliability to intensive longitudinal data with the Kalman filter

**DOI:** 10.1111/bmsp.70039

**Published:** 2026-02-18

**Authors:** Michael D. Hunter

**Affiliations:** ^1^ Department of Human Development and Family Studies Pennsylvania State University University Park Pennsylvania USA

**Keywords:** intensive longitudinal data, McDonald's ω, reliability, State Space Model, time series

## Abstract

Reliability is central to how researchers approach measurement in standard, group‐based analyses of single‐time‐point data, yet this critical aspect is often overlooked in the analysis of repeated observations. Since its inception, reliability has been a between‐person concept, but we redevelop this notion for within‐person designs by proposing a new coefficient κ of reliability for single‐subject designs. This coefficient shares the same general definition of reliability as former coefficients—the ratio of the true score variance to the total variance—but applies to time‐dependent within‐person variability rather than independent between‐person variability. Coefficient κ begins with a latent variable time series model called a state space model, and is then extended to a state space model for multiple subjects with continuous or discrete variation across people. Using analytic methods, we derive coefficient κ and prove its relations to other coefficients of reliability.

## 1 INTRODUCTION

Reliability is at the core of how researchers approach measurement in standard, group‐based analyses of single‐time‐point data. No established measure of differences between people lacks initial studies or reporting on its measurement characteristics. Coefficients of reliability and different aspects of validity are near universally reported when creating new measures of individual differences. Indeed, numerous books are written on the best ways to design and evaluate new measures to optimize measurement quality as assessed by these characteristics.

In spite of the near omnipresence of measurement assessment for single‐time‐point data, comparatively little focus has been given to multiple‐time‐point data in either single‐subject or multi‐subject designs. One reason for this neglect is certainly the historical paucity of intensive longitudinal data (ILD) in social and behavioural sciences. With the increased interest in person‐specific processes—and ILD more broadly—there has been a corresponding increase in approaches to assessments of measurement quality, resulting in a patchwork of partially overlapping approaches to the assessment of measurement quality when the individual is the unit of analysis (see Nezlek, 2017; Castro‐Alvarez et al., 2024, 2025, for recent reviews and tutorials). This patchwork demands a new foundation.

The absence of the necessary statistical foundations for within‐person measurement has persisted for long enough. Researchers first need clear ways to understand what measurement means at the individual level; they need a core set of measurement concepts applicable to the individual. Second, researchers need these concept to be translated into a mathematical framework and set of equations suitable to individual measurement. Finally, this mathematical framework needs to be available in statistical software. Of course, beyond meeting the needs of single‐subject and ILD researchers, an ideal measurement framework for the individual would have continuity with that already developed for groups of individuals. The present work aims to meet all of these needs. First, we generalize reliability to *within‐person* processes. Second, we extend within‐person reliability to multi‐subject time series which include single‐time‐point designs and longitudinal designs as special cases.

The structure of this paper is as follows. We begin with single‐subject data and develop a notion of reliability analogous to conventional, between‐person reliability for data that are independent over time. We then extend this within‐person reliability to general, time‐dependent data. Finally, we expand within‐person reliability to multi‐subject data, allowing for either continuous variation or discrete variation across subjects.

## 2 SINGLE‐SUBJECT RELIABILITY

Consider a single‐subject design aimed at the assessment of measurement quality for a new measure with m observed items. We use multiple items to cover the various aspects of a broad construct, knowing that some items might be more closely related to the construct than others and that our single‐subject might tend to endorse some items more than others. For the moment, let us assume that we administer the same set of items to a single person multiple times and that these repeated observations are independent over time. Lord and Novick (1968) begin their work in a very similar way. We can create a distribution of scores for each item across time. If we are willing to assume that the item scores come from a multivariate Gaussian distribution, then a factor model along the lines of Cattell's P‐technique factor analysis (Anderson, 1963; Cattell et al., 1947; Molenaar & Nesselroade, 2009; Nesselroade & Ford, 1985) would be a logical tool to understand the measurement structure and quality of our new measure. We can notate our P‐technique factor analysis as follows:

(1) $$f_{t}=k+q_{t}$$

(2) $$y_{t}=a+Lf_{t}+e_{t}$$

where $f_{t}$ is a vector of latent factors at time t, k is a vector of latent intercepts, $q_{t}$ is a vector of unmeasured disturbances at time t that directly impact the latent factors, $y_{t}$ is a vector of observed scores at time t, a is a vector of observed score intercepts, L is a matrix of factor loadings, and $e_{t}$ is a vector of unmeasured disturbances at time t that directly impact the observed scores. We assume (1) that $q_{t}$ is independent over time with mean zero and covariance matrix Q, (2) that $e_{t}$ is independent over time with mean zero and covariance matrix E, and (3) that $q_{t}$ and $e_{t}$ do not covary.

In a psychometric context, $f_{t}$ is often one‐dimensional—implying that k and $q_{t}$ are also one‐dimensional—but there can be utility in allowing $f_{t}$ to include multiple latent variables. Similarly, we let $y_{t}$ be M‐dimensional for a measurement instrument with M items, implying a and $e_{t}$ are also M‐dimensional. In the case of a single latent dimension, L becomes an M×1 matrix, but is generally an M×p matrix for p latent variables.

The above P‐technique factor model is the starting point for all subsequent considerations of reliability. Many coefficients of reliability follow directly from special cases of this model, and the main contribution of the present work follows from a slight generalization of this model. Throughout the present work we consider the variability of the true scores to be equal to the variability due to the latent factors $f_{t}$, and the variability of the observed scores to be that of $y_{t}$. Equation (3) makes this distinction between true score variance and observed score variance mathematically explicit in terms of the model‐implied covariance of the factor model in Equations (1) and (2):

(3) $$\underset{\text{Observed Score Variance}}{\underbrace{C}}=\underset{\text{True Score Variance}}{\underbrace{LQL^{\text{T}}}}+\underset{\text{Error Score Variance}}{\underbrace{E}}$$

As is common, we refer to the observed, true and error score variances even though these are all full variance–covariance matrices. For completeness, we also show the model‐implied means from Equations (1)and (2):

(4) m=a+Lk

Note that in the single‐subject and time‐independent case neither the model‐implied means nor the model‐implied covariances vary over time.

From this point, we can apply all the standard methods of classical test theory to obtain a modification to classical test theory that applies strictly to a single‐subject design. Such single‐subject designs are the natural starting place for a science of the individual (Nesselroade, 2010). Once a single‐subject reliability is defined, it can be extended to virtually any form of multi‐subject design. Using classical test theory in a single‐subject design, we can have a distribution of observed scores with a mean and a variance. The mean of the distribution of observed scores for a single subject is an estimate of the true score, as in classical test theory. Similarly, we can define reliability as the ratio of the true score variance to the observed score variance. In general, all the concepts from classical test theory immediately apply to single‐subject designs. We can define the Guttman–Cronbach (Cronbach, 1951; Guttman, 1945) α as

(5) $$\alpha =\frac{M}{M-1}\left(1-\frac{s^{\text{T}}\text{diag}\left(C\right)s}{s^{\text{T}}Cs}\right)$$

where M is the number of non‐zero weights in the scoring vector s, C is the model‐implied covariance matrix, the superscript T is the transpose operator, and diag(·) creates a diagonal matrix from the diagonal elements of a square matrix. Often, M is the total number of items and the scoring vector is equal to a vector of all 1s such that s=1. Appendix A shows several alternative formulas for α and shows they are equivalent to Equation (5).

Under certain circumstances, α is the appropriate definition of reliability. In particular, a 1‐factor model that has all loadings equal to one another L=k1, any factor variance $Q=\sigma_{q}^{2}$, and residual covariance matrix equal to a scalar multiple of the identity matrix $E=\sigma_{e}^{2}I$ will produce α as the coefficient of reliability. When measurement has equal loadings L=k1, it is called τ‐equivalent. The scalar structure of the residual covariance implies the residuals are independent and identically distributed. Thus, we have Proposition 1.

Proposition 1 *A 1‐factor model with* τ *‐equivalent measurement along with independent and identically distributed residuals yields* α *in Equation* *5* *as the definition of reliability.*

Appendix C provides a proof of Proposition 1.

Similar to the Guttman‐Cronbach α, we can define McDonald's (McDonald, 1970, 1999) ω for the P‐technique factor model as Equation (6)

(6) $$\omega =\frac{s^{\text{T}}LQL^{\text{T}}s}{s^{\text{T}}\left(LQL^{\text{T}}+E\right)s}=\frac{s^{\text{T}}LQL^{\text{T}}s}{s^{\text{T}}Cs}$$

where C is as defined by Equation (3). Equation (6) is not the typical form of the definition of ω; it is a generalization of ω for arbitrarily many factors of any structure (L and Q), a general residual covariance matrix (E), and any item‐weighting scheme (s). Conceptually, this definition of ω is the ratio of the weighted sum of the variance–covariance due to the latent variables to the weighted sum of the full, model‐implied covariance. That is, the ratio of the true score variance to the total variance, matching the definition of reliability.

For the special case of a single‐factor model with unit‐weighted sum s=1 and diagonal residual covariance matrix, Equation (6) is equivalent to the conventional definition of ω in Equation (7)

(7) $$\omega =\frac{{\left(\overset{M}{\underset{i=1}{\sum }}L_{i}\right)}^{2}\sigma_{q}^{2}}{{\left(\overset{M}{\underset{i=1}{\sum }}L_{i}\right)}^{2}\sigma_{q}^{2}+\overset{M}{\underset{i=1}{\sum }}E_{ii}}$$

Appendix D proves the equivalence of Equations (6) and (7) for a 1‐factor model with $Q=\sigma_{q}^{2}$, s=1, and E a diagonal matrix. Because a model matching this description is called a congeneric factor model, we have Proposition 2.

Proposition 2 *A 1‐factor model with congeneric measurement, along with independent distributed residuals, yields* ω *in Equation* *7* *as the definition of reliability.*

The proof of Proposition 2 is in Appendix D. Finally, McDonald (1999, p. 93) showed that ω≥α with equality in the case of τ‐equivalent measurement.

Proposition 3 *For a 1‐factor model with congeneric measurement along with independent distributed residuals,* ω *in Equation* *7* *is greater than or equal to* α *in Equation* *5* *, with equality holding if and only if the variance of factor loadings* L *is zero.*

All of the above Propositions and definitions are closely aligned with those usually reported for multi‐subject designs at a single time point. The fundamental difference is merely in the interpretation. The index in Equations (1) and (2) is t, referring to time points; however, because of the independence assumption over time, the t indexing times could easily be exchanged for i indexing independent subjects without the need to change any of the underlying mathematics. In this sense, the above descriptions are not novel; they merely change conventional findings from multi‐subject R‐technique factor analysis to single‐subject P‐technique factor analysis. The next section relaxes the assumption of independence over time and thus begins the novel contribution of the present work in earnest.

### 2.1 Dependence over Time

Up to this point, we have assumed independence of the observations over time. In their foundational book on the classical test theory, Lord and Novick (1968, p. 32) abandoned single‐subject designs and within‐person reliability at least in part due to difficulties with handling dependence over time. However, outside of test theory, a natural and general way of framing dependence over time already existed in the time series and engineering literature. The general way to model latent variables that change over time in quite flexible ways along with a measurement model is called a state space model. Mathematically, the state space model can be expressed as

(8) $$f_{t}=k+Af_{t-1}+q_{t}$$

(9) $$y_{t}=a+Lf_{t}+e_{t}$$

(10) $$f_{0}∼𝒩\left(k_{0},\;P_{0}\right)$$

where $f_{t}$ is a vector of latent factors at time t, k is a vector of latent intercepts, A is a matrix of autoregressive coefficients that map how the latent factors change over time, $q_{t}$ is a vector of unmeasured disturbances at time t that directly impact the latent factors, $y_{t}$ is a vector of observed scores at time t, a is a vector of observed score intercepts, L is a matrix of factor loadings, $e_{t}$ is a vector of unmeasured disturbances at time t that directly impact the observed scores, $k_{0}$ is the initial mean of the latent factors, and $P_{0}$ is the initial covariance of the latent factors. We assume the same properties for $q_{t}$ and $e_{t}$ as in the P‐technique factor model. Note that for simplicity we omit temporal subscripts on the model matrices and vectors (A, Q, E, L, k and a). This assumption is not strictly required for the vast majority of the following development, and we discuss relaxing it in detail later.

A careful comparison of Equations (2) and (9) reveals that these are, in fact, the same equation. Moreover, Equations (1) and (8) are nearly equal, except for the addition of the autoregressive term in Equation (8) that maps the latent factors at one time forward to the next time. Thus, the state space model can be thought of as a P‐technique factor analysis with autoregressive relations on the latent factors. Although the state space model in Equations ((8), (9), (10)) does not contain lagged factor loadings (cf., Molenaar, 1985, 2017), it is generally appropriate to call it a dynamic factor model.

Although the general form of the state space model appears to only account for lag‐1 autoregressive effects, it is sufficiently flexible to account for quite general changes over time, including arbitrarily many lags. In the standard time series modelling representation of change over time, a general vector autoregressive, integrated, moving average model (VARIMA) fits within this state space modelling framework (Hamilton, 1994a, 1994b; Shumway & Stoffer, 2010). Similarly, the set of models usually referred to as structural time series models (Harvey, 1989)—including models with seasonality and stochastic trends—fit within the state space modelling framework. Even conventional linear mixed effects models (Laird & Ware, 1982) and latent growth curve models (McArdle & Epstein, 1987) are special kinds of state space models (Jones, 1993). Thus, although the model in Equations ((8), (9), (10)) appears to assume that the disturbances on the latent factors and the observed scores ($q_{t}$ and $e_{t}$, respectively) are independent over time given lag‐1 autoregressive effects, in reality these equations assume that the dynamics in A are ‘parameterized sufficiently richly’ (e.g., Hamilton, 1994b, p. 327) to account for all temporal dependence^1^. Hence, the present work uses the state space model as the foundation for general within‐person modelling of change over time.

Unlike the P‐technique factor model which has a factor mean k and covariance Q that do not change over time, the factors of a state space model have a time‐varying mean and covariance structure (see Chow et al., 2010; Hunter, 2018, for introductions based on structural equation modeling). Because the emphasis of the present work is on reliability, we focus on the time‐varying covariance structure given by

(11) $$P_{t}=AP_{t-1}A^{\text{T}}+Q$$

where $P_{t}$ is the predicted covariance matrix of the latent factors at time t. This factor covariance is a function of the previous factor covariance $P_{t-1}$, the autoregressive dynamics A, and the covariance of the unmeasured, time‐varying disturbances that directly impact the latent factors Q. The factor covariance is initialized with $P_{0}$. In the single‐subject modelling case, $P_{0}$ is often chosen either as a diffuse prior (e.g. a scalar multiple of the identity matrix to allow for large variances) or as the stationary covariance matrix in Equation (13) to be discussed shortly.

The contribution of Kalman's (1960) landmark work was not in proposing the state space model, but rather in developing the recursive way to alternate steps between forecasting from a theoretical model and updating forecasts based on observed data. The Kalman filter optimally combines information from the prediction model and the observed data at each time point such that the imprecision of the forecast model and of the observations are both accommodated.

The covariance structure of the latent factors in turn induces a time‐varying covariance structure for the observed scores:

(12) $$C_{t}=LP_{t}L^{\text{T}}+E$$

But asymptotically over a large number of time points, the factor variance is

(13) $$\text{vec}(P_{\infty })={\left(I-A⊗A\right)}^{-1}\text{vec}(Q)$$

where vec() is the full vectorization operator that turns a matrix into a vector by concatenating all of its elements, I is a $p^{2}\times p^{2}$ identity matrix, and ⊗ is the Kronecker product. Equation (13) is the stationary covariance matrix for the latent factors. That is, the covariance matrix of the latent factors asymptotically approaches $P_{\infty }$ as the number of time points goes to infinity. The asymptotic covariance matrix for the latent factors allows for a parallel definition of the asymptotic variance of the observed scores

(14) $$C_{\infty }=LP_{\infty }L^{\text{T}}+E.$$

The covariances in Equations (12) and (14) allow for the definition of within‐person reliability with time‐dependent observations. We propose coefficient κ, named in honour of Rudolf Kalman, for the reliability of single‐subject measurement. Coefficient κ maintains the core foundation of reliability as the ratio of true score variability to total score variability while focusing on the individual and allowing that individual to change over time in extremely diverse ways. To allow for both stationary and non‐stationary processes, we actually propose two such coefficients. The first is the asymptotic, stationary reliability.

(15) $$\kappa_{\infty }=\frac{s^{\text{T}}LP_{\infty }L^{\text{T}}s}{s^{\text{T}}C_{\infty }s}$$

Equation (15) gives the ratio of the variance of the latent factors to the observed scores asymptotically as the number of time points goes to infinity. For stationary processes $\kappa_{\infty }$ summarizes the reliability of a single‐subject design in a single number, much the same as α and ω do when the process is independent over time. However, some processes of interest are not stationary. So, we propose the second coefficient in Equation (16), giving a time‐varying coefficient of reliability that does not assume the process is stationary:

(16) $$\kappa_{t}=\frac{s^{\text{T}}LP_{t}L^{\text{T}}s}{s^{\text{T}}C_{t}s}$$

Equation (16) replaces the asymptotic covariance of the latent factors, $P_{\infty }$, with the time‐varying covariance of the latent factors, $P_{t}$. Thus, there is potentially a different reliability for every time point. It is reasonable for the reliability of a non‐stationary process to not be summarized by a single number. By definition, the means and covariances of non‐stationary processes change over time. Thus, the ratio of the true score variability to the observed score variability is expected to be time‐varying.

The main difference between ω and κ is accounting for the dynamic relations among the factors. From Equation (11), it is evident that if the factors were independent over time (i.e., if A=0), then the total factor covariance $P_{t}$ would equal the covariance of the unmeasured disturbances at any given time Q. Simply put, if there are no dynamics, then $P_{t}=P_{\infty }=Q$. Coefficient κ is a way of accounting for the time‐dependence of the covariance of the latent factors. With that general understanding, an important question remains: how are ω and κ related? Proposition 4 addresses this question.

Proposition 4 *For a 1‐factor model with congeneric measurement,* $\kappa_{\infty }$ *in Equation* *15* *equals or exceeds* ω *in Equation* *6* *.*

Appendix F provides a proof of Proposition 4.

Figure 1 guides our understanding of the relation between ω and $\kappa_{\infty }$ further by showing how κ varies as a function of the autoregressive coefficient, A, in 1‐factor models. When A=0, ω and κ are equal. At all other points where the dynamics are stationary, $\kappa_{\infty }>\omega$. Outside this stationary range, $\kappa_{\infty }$ gives a non‐meaningful reliability: greater than 1, or less than zero.

**FIGURE 1:**
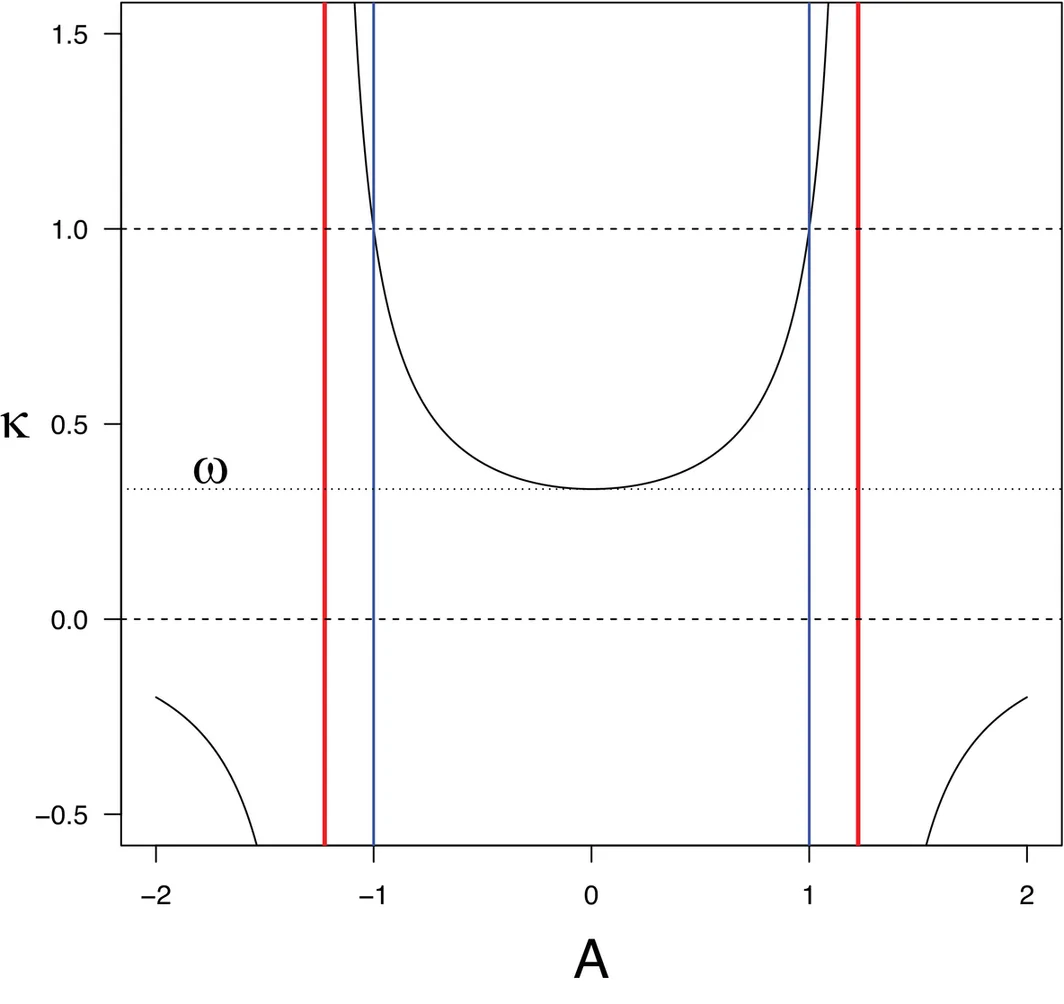
Example relation between $\kappa_{\infty }$ and ω for a dynamic 1‐factor model as a function of the autoregressive coefficient A. The solid black lines give the value of κ, the reciprocal of a quadratic function of A. The dotted horizontal black line gives the value of ω. The dashed horizontal black lines give the theoretical range of a reliability coefficient [0, 1]. The vertical blue lines give the boundaries for stable autoregressive coefficients [−1, 1]. The vertical red lines give the roots of the quadratic function where κ is undefined. The time‐independent signal‐to‐noise ratio in this example is .5.

The functional form for $\kappa_{\infty }$ seen in Figure 1 is made more intuitive by restating the formula for $\kappa_{\infty }$ as a function of the autoregressive coefficient A in the 1‐factor case.

(17) $$\kappa_{\infty }=-SNR_{\omega }\frac{1}{A^{2}-1-SNR_{\omega }}$$

where $SNR_{\omega }$ is the signal‐to‐noise ratio from ω and is given by Equation (18).

(18) $$SNR_{\omega }=\frac{s^{\text{T}}LQL^{\text{T}}s}{s^{\text{T}}Es}$$

Thus, in the 1‐factor case, $\kappa_{\infty }$ is the reciprocal of a quadratic polynomial in the autoregressive coefficient, A.

### 2.2 Further considerations for non‐stationary processes

The previous development has assumed that although there may be a time‐varying latent process as described by Equations (8–10), the rules that govern this process are time‐invariant. However, this time‐invariant assumption is merely a conceptual convenience rather than a mathematical necessity. The rules that govern the process could be time‐varying. That is, the matrices A, Q, E and L along with the vectors k and a could include a subscript t. If A, Q or k vary over time, then the latent process is non‐stationary, changing how the process works as a function of time. If E, L or a vary over time, then there is measurement non‐invariance over time (see Meredith & Horn, 2001, for a general discussion). Of course, this measurement non‐invariance is also a kind of non‐stationarity.

Although a full discussion of time‐varying dynamics and measurement is beyond the scope of the present work, it has been a mainstay of psychological and developmental research for more than half a century. Baltes and Nesselroade (1970) proposed separating developmental change from generational differences by examining differences in both factor loadings and factor scores in appropriate research designs. Later, Olsson and Bergman (1977) allowed temporal measurement non‐invariance in a two‐time‐point longitudinal factor model of cognitive abilities and achievement. More recently, Molenaar et al. (2009) allowed factor loadings (L) and autoregressive coefficients (A) to follow time‐varying, stochastic random walks (equation 2, p. 264). Even more recently, methodological developments in ‘dynamic structural equation modelling’ have also allowed for time‐varying factor loadings (McNeish et al., 2021). Moreover, regime‐switching mixture models can let measurement and/or dynamic structures differ across persons (Hunter, 2014, 2024) and contexts (Vogelsmeier et al., 2024).

With all of these various forms of time‐varying models, it is important to consider whether the meaning of the factor is conceptually consistent in spite of the violation of measurement invariance. Nesselroade and Molenaar (2016) argued that—at least in some cases—the meaning of a factor can persist in the presence of time‐varying factor loadings. Regardless of the form of time‐varying model, the practical solution for a time‐varying reliability coefficient is simple: use $\kappa_{t}$ as defined by Equation (16) instead of $\kappa_{\infty }$ as defined by Equation (15). However, we must urge researchers when using $\kappa_{t}$ to provide some evidence for the permissibility of invoking a time‐varying reliability coefficient. There are many conceptual challenges surrounding validity and reliability of factors in time‐varying models, some of which may obviate the notion of reliability.

### 2.3 Summary of key relations for single‐subject reliability

At this point, it is prudent to summarize and synthesize the key results shown. For completeness, we restate some definitions given previously. By a ‘factor model’ we mean a model represented by Equations (1) and (2). By a ‘dynamic factor model’ we mean a model represented by Equations ((8), (9), (10)). By ‘congeneric measurement’ we mean a generic arrangement of factor loadings L. By ‘τ‐equivalent’ measurement we mean L=k1. By ‘independently and identically distributed residuals’ we mean $E=\sigma_{e}^{2}I$. Finally, by ‘independently distributed residuals’ we mean E is a diagonal matrix.

Proposition 5 *Suppose a dynamic* p *‐factor model has congeneric measurement and zero autoregression coefficient, then* $\omega =\kappa_{\infty }=\kappa_{t}$ *.*

If A=0, then $P_{\infty }=P_{t}=Q$. It then follows that $\kappa_{\infty }=\kappa_{t}=\omega$ for any scoring method (s) and any factor structure (L, Q and E). Coefficients $\kappa_{t}$ and $\kappa_{\infty }$ both generalize ω to time‐dependent, single‐subject models.

Combining Propositions 5 and the *equality* case of Proposition 3 we have the following:

Proposition 6 *Suppose a 1‐factor model has* τ *‐equivalent measurement and independently and identically distributed residuals, then* $\alpha =\omega =\kappa_{t}=\kappa_{\infty }$ *.*

Relatedly, combing Proposition 5 and the *general* case of Proposition 3 yields the following:

Proposition 7 *Suppose a 1‐factor model has congeneric measurement and independently distributed residuals, then* $\alpha \leq \omega =\kappa_{t}=\kappa_{\infty }$ *.*

Finally, using Proposition 4 and 3 results in the primary result of the present work.

Proposition 8 *Suppose a dynamic 1‐factor model has congeneric measurement, a stationary autoregressive coefficient* |A|<1 *, and independently distributed residuals, then* $\alpha \leq \omega \leq \kappa_{\infty }$ *. In this case,* α=ω *when the factor loadings are constant (i.e.* τ *‐equivalent). Also,* $\omega =\kappa_{\infty }$ *when the dynamics are zero.*

## 3 MULTI‐SUBJECT RELIABILITY

The previous sections have developed a notion of reliability and a set of coefficients for reliability that apply to single‐subject designs. Although the single‐subject design is a logical place to begin thinking about measurement and reliability, the vast majority of research designs in social and behavioural sciences involve multiple subjects. Some of these research designs involve multiple time points, but others entail a single occasion of measurement. Moreover, among the multi‐subject designs that also involve multiple time points, some assume that all subjects are homogeneous, whereas others allow for different kinds of heterogeneity across subjects. We address all of these situations.

### 3.1 Homogeneous subjects

In all single‐time‐point designs, we assume that subjects are homogeneous, at least after controlling for some set of covariates, or allowing for some number of unmeasured groups (e.g., via mixture modelling). Even in many multiple‐time‐point designs, it is convenient to assume that individuals are homogeneous after controlling for covariates or underlying groups. We consider both the single‐time‐point and multiple‐time‐point designs as a special case of the multi‐subject state space model.

(19) $$f_{i,t}=k+Af_{i,t-1}+q_{i,t}$$

(20) $$y_{i,t}=a+Lf_{i,t}+e_{i,t}$$

(21) $$f_{i,0}∼𝒩\left(k_{0},\;P_{0}\right)$$

All the terms in Equations ((19), (20), (21)) have a similar conceptual and mathematical meaning to those discussed in Equations ((8), (9), (10)), but now many of the terms also vary over subject i in addition to over time point t. Note that the homogeneity assumption appears in that the dynamics A, factor loadings L, latent factor disturbances Q and observed variable disturbances E do not depend on person i and are thus homogeneous across people.

In the special case of the multi‐subject and single‐time‐point design, it is no longer possible to estimate the dynamics A which must be fixed to an identity matrix. Similarly, the across‐time intercept k and the latent disturbance covariance Q must be fixed to a zero vector and zero matrix, respectively, but the other aspects of the model remain intact. The initial conditions $k_{0}$ and $P_{0}$ take on a new meaning as the between‐person (i.e., ensemble) mean and covariance for the latent factors. This scenario becomes exactly equivalent to a typical R‐technique factor model. The $\kappa_{t}$ and ω coefficients of reliability must use the between‐person covariance structure $P_{0}$ instead of $P_{t}$ or Q in their definitions. Consequently, their interpretation must change to the reliability of between‐person differences rather than the reliability of within‐person differences, but their mathematical form is preserved. Because these coefficients now represent between‐person variability, they are renamed with the subscript b.

(22) $$\kappa_{b}=\omega_{b}=\frac{s^{\text{T}}LP_{0}L^{\text{T}}s}{s^{\text{T}}C_{0}s}$$

where $C_{0}$ is defined analogously as in Equations (12) and (14): the total predicted covariance for the observed variables based on the between‐person covariance structure $P_{0}$. Notably, for the single‐time‐point, multiple‐subject state space model, the initial covariance structure does not change over time. Thus, $P_{0}=P_{t}=P_{\infty }$ and therefore $C_{0}=C_{t}=C_{\infty }$. Finally, it follows that $\kappa_{t}=\kappa_{\infty }=\kappa_{b}$ as well.

In the general case of the multi‐subject and multiple‐time‐point design, the original definitions of $\kappa_{t}$ and ω are valid with the same interpretation as within‐person reliability. Critically, however, this interpretation now rests on the assumption of homogeneity across subjects. Several authors have strongly argued against the plausibility of this homogeneity assumption (Fisher et al., 2018; Hunter et al., 2024; Molenaar, 2004; Nesselroade & Molenaar, 2016). In addition to the standard within‐person reliability a between‐person variability becomes possible in these multi‐subject and multiple‐time‐point designs. If the variation between people is assumed to be continuous and Gaussian, then we have the kind of multilevel reliability considered next.

### 3.2 Continuously varying groups

Lai (2021) gave a set of equations for the reliability in a multivariate, multilevel factor model. The within‐cluster reliability was given as $\omega_{w}$.

(23) $$\omega_{w}=\frac{1^{\text{T}}\Sigma_{w}1-1^{\text{T}}E_{w}1}{1^{\text{T}}\Sigma_{w}1}=\frac{1^{\text{T}}L_{w}Q_{w}L_{w}^{\text{T}}1}{1^{\text{T}}\Sigma_{w}1}$$

where $\Sigma_{w}$ is the within‐cluster covariance matrix given by a within‐cluster factor analysis.

(24) $$\Sigma_{w}=L_{w}Q_{w}L_{w}^{\text{T}}+E_{w}$$

where $L_{w}$, $Q_{w}$ and $E_{w}$ form the within‐cluster factor model.

By analogy with univariate multilevel reliability, Lai (2021) gave the between‐cluster reliability as

(25) $$\omega_{b}=\frac{1^{\text{T}}\Sigma_{b}1-1^{\text{T}}E_{b}1}{1^{\text{T}}\Sigma_{b}1+1^{\text{T}}\Sigma_{w}1/\tilde{T}}$$

where $\Sigma_{b}$ is the between‐cluster covariance matrix given by a between‐cluster factor analysis

(26) $$\Sigma_{b}=L_{b}Q_{b}L_{b}^{\text{T}}+E_{b}$$

and $\tilde{T}$ is the harmonic mean of the number of level‐1 units per level‐2 unit.

For the purposes of the present work, the within‐cluster variation is across time, and the between‐cluster variation is across people. Hence, $\omega_{w}$ gives the within‐person reliability, $\omega_{b}$ gives the between‐person reliability, and $\tilde{T}$ is the harmonic mean of the number of time points per person.

If we substitute the time‐varying covariance matrix of the latent factors from a state space model into $\Sigma_{w}$, we have a non‐stationary, multilevel coefficient of reliability.

(27) $$\kappa_{w,t}=\frac{s^{\text{T}}C_{t}s-s^{\text{T}}Es}{s^{\text{T}}C_{t}s}=\frac{s^{\text{T}}LP_{t}L^{\text{T}}s}{s^{\text{T}}C_{t}s}$$

If we further add factor structure to the between‐person covariance from the multi‐subject state space model such that $\Sigma_{b}=L_{b}Q_{b}L_{b}^{\text{T}}+E_{b}$, we have a non‐stationary between‐person coefficient of reliability:

(28) $$\kappa_{b,t}=\frac{s^{\text{T}}\Sigma_{b}s-s^{\text{T}}E_{b}s}{s^{\text{T}}\Sigma_{b}s+s^{\text{T}}C_{t}s^{\text{T}}/\tilde{T}}$$

Replacing $C_{t}$ and $P_{t}$ with $C_{\infty }$ and $P_{\infty }$ in the above equations turns them into the stationary versions of multilevel κ.

### 3.3 Discretely varying groups

In addition to the possibility that variation between people is continuous, it is worthwhile to consider the possibility that between‐person variation is discrete. Hunter (2014, 2024) developed such a method of modelling discrete variation across people while simultaneously representing each individual by a state space model. State space mixture modelling combines mixture models for between‐person variation with state space models for within‐person variation over time. State space mixture modelling is Gaussian mixture modelling where each mixture class is a multi‐subject state space model. Importantly, the mixtures operate at the person‐level, rather than the time‐level. Consequently, the reliability within each mixture class is merely the standard $\kappa_{\infty }$ or $\kappa_{t}$ from Equations (15) and (16), respectively.

## 4 RELATION OF κ TO FURTHER RELIABILITY COEFFICIENTS

Castro‐Alvarez et al. (2024) conducted an extensive and in‐depth review of reliability coefficients applied to intensive longitudinal data (ILD). In this review, numerous methods for computing reliability were described and applied to ILD, including (1) generalizability theory (G‐theory; Cronbach et al., 1963; Cranford et al., 2006), (2) multilevel regression (Nezlek, 2017), (3) multilevel confirmatory factor analysis (Lai, 2021), (4) P‐technique factor analysis (Cattell et al., 1947), (5) dynamic factor analysis (Browne & Nesselroade, 2005), (6) multilevel dynamic factor analysis (Asparouhov et al., 2017; Xiao et al., 2023) and (7) latent state‐trait theory (Castro‐Alvarez et al., 2022; Geiser et al., 2014; Steyer et al., 1999, 2015). Although a full, mathematical description and proof is beyond the current space limitations, it is possible to consider all of these methods for computing reliability as special cases of the current framework for κ.

The current reliability framework takes as axiomatic the definition of reliability as the ratio of the true score variance to the total variance, but then allows for a wide variety of ways to conceptualize what is counted as true score variance and what is counted as total variance. The framework allows for multiple latent variables, differential item weights with factor loadings, a general model of dependence over time, and multiple, possibly heterogeneous subjects which can vary continuously or discretely. From this overarching framework, the Guttman‐Cronbach α coefficient is a model‐based instantiation of the definition of reliability under a model that has a single factor, no dependence over observations, equal factor loadings and independent and identically distributed residuals across items (Proposition 1). Under a more relaxed factor model, McDonald's ω coefficient is a model‐based instantiation of the definition of reliability (Proposition 2). If the data are repeated, time‐independent observations on a single subject, then McDonald's ω is a suitable reliability coefficient for P‐technique factor analysis. The critical aspects of the framework are twofold: (1) the axiomatic definition of reliability as the ratio of true score variance to total variance and (2) a suitable model for true scores and total scores. Different models instantiate different coefficients of reliability only to the extent that they define true score and total score variance differently; the different models lead to different reliability coefficients. This framework sheds light on the many reliability coefficients appropriate for ILD.

There are many coefficients of reliability for ILD because there are many possible appropriate models for ILD. G‐theory makes a random effects analysis of variance (ANOVA) model for ILD and its various generalizability coefficients result from varying considerations about which of those random effects contribute to the true score variance and the total variance. The multilevel confirmatory factor analysis makes a different model for ILD and results in a reliability coefficient that is often distinct from that of G‐theory. Similarly, each reliability coefficient considered by Castro‐Alvarez et al. (2024) results from a different model. However, the framework here presented for coefficient κ is the state space model which includes the vast majority of previously‐considered models for ILD as special cases. The random effects ANOVA model of G‐theory is a special case of multilevel regression which in turn is a special case of the state space model (Jones, 1993). Likewise, the multilevel random intercepts confirmatory factor model considered by Lai (2021) is a special case of the state space model (Gu et al., 2014). Both P‐technique factor analysis and dynamic factor analysis are quite straightforwardly particular kinds of state space models (Molenaar, 1985). Furthermore, latent state‐trait theory is often implemented as a structural equation model (e.g., Castro‐Alvarez et al., 2022), and structural equation models are state space models with zero dynamics over time (Hunter, 2018, equations 13–15)^2^. Finally, multilevel dynamic factor analysis can be thought of as a state space model with random effects by person allowed on all parameters and estimated with Bayesian methods. A version of this model was considered under the heading of ‘Continuously Varying Groups’ above. So, in this abstract sense, the framework that leads to coefficient κ is sufficiently broad to cover a wide variety of seemingly different reliability coefficients. In harmony with Savalei and Reise (2019), we emphasize the importance of model development for ILD such that the most appropriate instance of κ will result from the most appropriate model.

## 5 DISCUSSION

The core of reliability is the ratio of true score variance to total variance. This ratio is of central concern for measurement in the social and behavioural sciences. However, the increasing emphasis on and availability of intensive longitudinal data (ILD) in psychology and related disciplines has thus far neglected this concern for the assessment of measurement quality. The difficulty of handling time‐dependent processes and their time‐varying variances has historically presented an impediment to the definition of reliability in these ILD designs. The present work sought to remove this impediment. To do so, we began with single‐subject designs before broadening the results to multi‐subject designs of arbitrarily many time points. Our primary contribution is the proposal for a coefficient κ of reliability for single‐subject designs, along with its several variations for multi‐subject designs appropriate to ILD.

Although novel, κ is built upon the same foundation as other measures of reliability. In single‐time‐point designs, we showed that κ is equal to McDonald's ω and to the Guttman‐Cronbach α for 1‐factor models with equal factor loadings and independent and identically distributed residuals. In general factor models for single‐time‐point designs, ω and κ are equal. In multiple‐time‐point designs for dynamic 1‐factor models, we showed that κ≥ω≥α. In general, κ directly extends ω to within‐person processes that are dependent over time. Finally, we showed how κ can be extended both to continuous variation between people in multilevel models and to discrete variation between people in mixture models.

With any coefficient resulting from the combination of estimated parameters from a model, statistical inference is an important matter. We presented ways to compute κ based on a model, but this model contains free parameters that are estimated with some non‐zero sampling variability. Thus, κ is not infinitely, mathematically precise, but rather has some sampling variability inherited from the free parameters. Because the various factor and state space models considered here can be run in the OpenMx (Neale et al., 2016) R (R Core Team, 2023) package with its general structural equation model facilities or its state space modelling facilities (Hunter, 2018), two forms of statistical inference are readily available. First, Wald‐type confidence intervals based on the standard errors of the free parameters and the delta method can give standard errors and confidence intervals for κ with the mxSE and confint.MxModel functions. Second, profile likelihood confidence intervals (Neale & Miller, 1997; Pritikin et al., 2017) for κ can be obtained from mxCI. These two method of statistical inference allow for best practices in reporting the coefficient of reliability along with a measure of its precision.

Despite these contributions, several issues remain unaddressed. First, there is a substantial literature on reliability in growth curve modelling (Laenen et al., 2008; Marcoulides, 2019; Willett, 1989). It is possible to fit a latent growth curve model as a multi‐subject state space model, and our initial explorations found identical results to the method suggested by Marcoulides (2019), but the general relation between κ and several growth curve modelling coefficients of reliability is not known. Neither is a strict relation known between κ and the within‐person standard deviation (cf. Du & Wang, 2018). Second, although κ extends to multiple factor models, we did not explore popular multiple factor structures like the bifactor model and the hierarchical ω coefficient (Li & Savalei, 2025). Third, we did not compare κ to other coefficients of reliability for vector autoregressive models with measurement error and between‐person differences (e.g., Schuurman & Hamaker, 2019). Fourth and finally, there are several tutorials on ways of handling reliability for ILD designs with previously existing methods (e.g., van Alphen et al., 2022). Concrete, worked example problems using κ would certainly help researchers apply this novel reliability coefficient to their data. These limitations notwithstanding, κ is a new tool in the assessment of measurement quality for repeated measures designs.

## AUTHOR CONTRIBUTIONS

**Michael D. Hunter:** conceptualization; writing – original draft; methodology; visualization; writing – review and editing; software; formal analysis; investigation; validation.

## DISCLOSURE OF ARTIFICIAL INTELLIGENCE‐GENERATED CONTENT

No generative AI tools were used in the creation of this article.

## Data Availability

Data sharing not applicable to this article as no datasets were generated or analysed during the current study.

## Footnotes

1 If in doubt, a set of regression‐style residual diagnostics could be employed to check this assumption (e.g., Wooldridge, 1991).

2 Note that many of these equivalences are actually bidirectional. For example, state space models—sometimes called linear systems theory—are also special cases of structural equation models (MacCallum & Ashby, 1986).

## APPENDIX A EQUATIONS FOR α

For M the number of items and C the covariance matrix of the items, the usual equation for the Guttman–Cronbach (Cronbach, 1951; Guttman, 1945) α is

(A1)
$$\alpha =\frac{M}{M-1}\left(1-\frac{\overset{M}{\underset{i=1}{\sum }}C_{ii}}{\overset{M}{\underset{i=1}{\sum }}\overset{M}{\underset{j=1}{\sum }}C_{ij}}\right)$$

However, it is often beneficial to be able to write this equation in a matrix form as

(A2)
$$\alpha =\frac{M}{M-1}\left(1-\frac{\text{tr}\left(C\right)}{1^{\text{T}}C1}\right)$$

where 1 is a vector of all 1s, and tr(·) is the trace of a matrix (i.e., the sum of the diagonal elements). A slightly further generalization replaces 1 by a scoring vector s of item weights. These item weights might be chosen to yield maximum reliability (Bentler, 1968), to evaluate a subscale score, to allow for multiple factors, to reverse code items or some combination of all of these. In the case of a more generic scoring vector, is it useful to redefine m not as the number of items in a scale, but as the number of non‐zero weights? That is, define $M:=\|s{\|}_{0}$, the 0‐norm of the scoring vector, that is, the number of non‐zero elements of s:

(A3)
$$\alpha =\frac{M}{M-1}\left(1-\frac{s^{\text{T}}\text{diag}\left(C\right)s}{s^{\text{T}}Cs}\right)$$

## APPENDIX B EQUATIONS FOR ω

For M the number of items, McDonald's (1970, 1999) ω coefficient of reliability is usually defined as

(B1)
$$\omega =\frac{{\left(\overset{M}{\underset{i=1}{\sum }}L_{i}\right)}^{2}\sigma_{q}^{2}}{{\left(\overset{M}{\underset{i=1}{\sum }}L_{i}\right)}^{2}\sigma_{q}^{2}+\overset{M}{\underset{i=1}{\sum }}E_{ii}}$$

However, it is often beneficial to write this equation in matrix form as

(B2)
$$\omega =\frac{1^{\text{T}}LQL^{\text{T}}1}{1^{\text{T}}\left(LQL^{\text{T}}+E\right)1}$$

The equivalence of Equation (B1) to Equation (B2) is shown in Appendix D.

Just as with α, coefficient ω can be broadened to a general set of scoring weights s instead of the unit‐weighted sum of the full scale with 1.

(B3)
$$\omega =\frac{s^{\text{T}}LQL^{\text{T}}s}{s^{\text{T}}\left(LQL^{\text{T}}+E\right)s}$$

## APPENDIX C DERIVATION OF α FROM A FACTOR MODEL

We derive the usual definition of α from a 1‐factor model with equal loadings L=k1 and independent and identically distributed residuals $E=\sigma_{e}^{2}I$. We begin with several equalities that will be used later.

First, we see that the sum of the true score variance is equal to the square of the factor loading times the factor variance times the square of the number of items.

(C1)
$$\text{sum}(LQL^{\text{T}})=1^{\text{T}}LQL^{\text{T}}1=1^{\text{T}}(k1)\sigma_{q}^{2}{\left(k1\right)}^{\text{T}}1=k^{2}\sigma_{q}^{2}(1^{\text{T}}1)(1^{\text{T}}1)=k^{2}\sigma_{q}^{2}(M)(M)=k^{2}\sigma_{q}^{2}M^{2}$$

Next, we restate the residual variance of each observed variable in terms of the factor loading, the factor variance and the total observed variance of each variable.

(C2)
$$C_{ii}=c=k^{2}\sigma_{q}^{2}+\sigma_{e}^{2}\Rightarrow \sigma_{e}^{2}=c-k^{2}\sigma_{q}^{2}$$

The sum of the diagonal elements of the covariance matrix of the observed variables can be stated quite simply.

(C3)
tr(C)=Mc

The sum of the full covariance matrix for the observed variables can also be stated simply.

(C4)
$$\begin{array}{ll} \text{sum}(C) & =1^{\text{T}}C1=1^{\text{T}}(LQL^{\text{T}}+E)1=1^{\text{T}}LQL^{\text{T}}1+1^{\text{T}}E1=k^{2}\sigma_{q}^{2}M^{2}+M\sigma_{e}^{2}\; \end{array}$$

(C5)
$$\begin{array}{ll} & =k^{2}\sigma_{q}^{2}M^{2}+M(c-k^{2}\sigma_{q}^{2})=k^{2}\sigma_{q}^{2}M^{2}+Mc-Mk^{2}\sigma_{q}^{2}\; \end{array}$$

(C6)
$$\begin{array}{ll} & =M(M-1)k^{2}\sigma_{q}^{2}+Mc\; \end{array}$$

Finally, the difference between the full sum and the sum of the diagonal elements of the covariance matrix for the observed variables can be stated separately.

(C7)
$$\text{sum}(C)-\text{tr}(C)=M(M-1)k^{2}\sigma_{q}^{2}+Mc-Mc=M(M-1)k^{2}\sigma_{q}^{2}$$

We now use the equalities shown above to derive α from this 1‐factor model.

(C8)
$$\begin{array}{ll} \frac{\text{sum}(LQL^{\text{T}})}{\text{sum}(C)} & =\frac{M(M-1)}{M(M-1)}\cdot \frac{\text{sum}(LQL^{\text{T}})}{\text{sum}(C)}=\frac{M(M-1)}{M(M-1)}\cdot \frac{k^{2}\sigma_{q}^{2}M^{2}}{\text{sum}(C)}\; \end{array}$$

(C9)
$$\begin{array}{ll} & =\frac{M^{2}}{M(M-1)}\cdot \frac{k^{2}\sigma_{q}^{2}M(M-1)}{\text{sum}(C)}=\frac{M^{2}}{M(M-1)}\cdot \frac{\text{sum}(C)-\text{tr}(C)}{\text{sum}(C)}\; \end{array}$$

(C10)
$$\begin{array}{ll} & =\frac{M^{2}}{M(M-1)}\cdot \left(1-\frac{\text{tr}(C)}{\text{sum}(C)}\right)=\frac{M}{M-1}\cdot \left(1-\frac{\text{tr}(C)}{\text{sum}(C)}\right)\; \end{array}$$

(C11)
$$\begin{array}{ll} & =\alpha \; \end{array}$$

## APPENDIX D DERIVATION OF ω FROM A FACTOR MODEL

We derive the typical equation for ω from a factor model as the ratio of a the variance due to the latent factors to the variance due to the observed scores.

(D1)
$$\begin{array}{ll} \frac{1^{\text{T}}LQL^{\text{T}}1}{1^{\text{T}}\left(LQL^{\text{T}}+E\right)1} & =\frac{1^{\text{T}}LL^{\text{T}}1\sigma_{q}^{2}}{1^{\text{T}}LL^{\text{T}}1\sigma_{q}^{2}+1^{\text{T}}E1}=\frac{\left(1^{\text{T}}L\right)\left(L^{\text{T}}1\right)\sigma_{q}^{2}}{\left(1^{\text{T}}L\right)\left(L^{\text{T}}1\right)\sigma_{q}^{2}+1^{\text{T}}E1}\; \end{array}$$

(D2)
$$\begin{array}{ll} & =\frac{\left(\overset{M}{\underset{i=1}{\sum }}L_{i}\right)\left(\overset{M}{\underset{i=1}{\sum }}L_{i}\right)\sigma_{q}^{2}}{\left(\overset{M}{\underset{i=1}{\sum }}L_{i}\right)\left(\overset{M}{\underset{i=1}{\sum }}L_{i}\right)\sigma_{q}^{2}+\overset{M}{\underset{i=1}{\sum }}E_{ii}}\; \end{array}$$

(D3)
$$\begin{array}{ll} & =\frac{{\left(\overset{M}{\underset{i=1}{\sum }}L_{i}\right)}^{2}\sigma_{q}^{2}}{{\left(\overset{M}{\underset{i=1}{\sum }}L_{i}\right)}^{2}\sigma_{q}^{2}+\overset{M}{\underset{i=1}{\sum }}E_{ii}}=\omega \; \end{array}$$

## APPENDIX E DERIVATION OF α FROM ω

We show that α can be derived from ω for a 1‐factor model with equal loadings L=k1 and independent and identically distributed residuals $E=\sigma_{e}^{2}I$.

(E1)
$$\omega =\frac{{\left(\overset{M}{\underset{i=1}{\sum }}L_{i}\right)}^{2}\sigma_{q}^{2}}{{\left(\overset{M}{\underset{i=1}{\sum }}L_{i}\right)}^{2}\sigma_{q}^{2}+\overset{M}{\underset{i=1}{\sum }}E_{ii}}=\frac{{\left(Mk\right)}^{2}\sigma_{q}^{2}}{{\left(Mk\right)}^{2}\sigma_{q}^{2}+M(c-k^{2}\sigma_{q}^{2})}=\frac{M^{2}k^{2}\sigma_{q}^{2}}{M^{2}k^{2}\sigma_{q}^{2}+Mc-Mk^{2}\sigma_{q}^{2}}$$

From this point, follow the steps and Equations at the beginning of Appendix C to yield α.

## APPENDIX F PROOF THAT κ MEETS OR EXCEEDS ω

We show that κ≥ω for 1‐factor models. As a preliminary step, we need a basic property of fractions. Let a, b and c be real numbers all greater than zero. Furthermore, let a≥b. We seek to show that under these conditions

(F1)
$$\frac{a}{a+c}\geq \frac{b}{b+c}$$

The proof proceeds as follows:

(F2)
$$\begin{array}{ll} a & \geq b\;\text{Given.}\; \end{array}$$

(F3)
$$\begin{array}{ll} ac & \geq bc\;\text{Multiplying both sides by}\;c\; \end{array}$$

(F4)
$$\begin{array}{ll} ab+ac & \geq ab+bc\;\text{Adding}\;ab\;\text{to both sides}\; \end{array}$$

(F5)
$$\begin{array}{ll} a(b+c) & \geq b(a+c)\;\text{Association}\; \end{array}$$

(F6)
$$\begin{array}{ll} \frac{a(b+c)}{\left(a+c)(b+c\right)} & \geq \frac{b(a+c)}{\left(a+c)(b+c\right)}\;\text{Dividing both sides by}\;(a+c)(b+c)\; \end{array}$$

(F7)
$$\begin{array}{ll} \left(\frac{a}{a+c}\right)\left(\frac{b+c}{b+c}\right) & \geq \left(\frac{b}{b+c}\right)\left(\frac{a+c}{a+c}\right)\;\text{Association}\; \end{array}$$

(F8)
$$\begin{array}{ll} \frac{a}{a+c}1 & \geq \frac{b}{b+c}1\;\text{Cancellation}\; \end{array}$$

(F9)
$$\begin{array}{ll} \frac{a}{a+c} & \geq \frac{b}{b+c}\;\text{Cancellation}\; \end{array}$$

We will use the above property at a later step.

We next show that the numerator of κ is greater than or equal to the numerator of ω. For a 1‐factor model, the general definition of the asymptotic latent variance–covariance matrix in Equation (13), reduces to

(F10)
$$P_{\infty }={\left(1-A⊗A\right)}^{-1}Q=\frac{Q}{1-A^{2}}$$

where the vec operator is no longer needed and the symbols are no longer bold because the matrices are all 1×1. Substituting Equation (F10) into the numerator of the 1‐factor definition of κ in Equation (15) yields

(F11)
$$s^{\text{T}}LP_{\infty }L^{\text{T}}s=s^{\text{T}}L\left(\frac{Q}{1-A^{2}}\right)L^{\text{T}}s=\left(\frac{1}{1-A^{2}}\right)s^{\text{T}}LQL^{\text{T}}s\geq s^{\text{T}}LQL^{\text{T}}s$$

where the inequality follows because $1/(1-A^{2})\geq 1$ for any |A|<1. So, the numerator for κ is greater than or equal to the numerator for ω.

Next, we show that the numerators and denominators of κ and ω are positive. Note that the numerators of both κ and ω are quadratic forms. If Q and $P_{\infty }$ are positive definite, then the numerators are positive. Q is positive definite (i.e., a scalar greater than zero), and $P_{\infty }$ is positive definite whenever |A|<1. The denominators of both κ and ω are sums of quadratic forms. The first part of both sums is the numerator which we have just shown is positive definite. The second part of both sums is $s^{\text{T}}Es$. This quadrative form is also positive definite because E is a covariance matrix. So, the numerators and denominators of κ and ω are positive.

If we call the numerator of κ by $a=s^{\text{T}}LP_{\infty }L^{\text{T}}s$, the numerator of ω by $b=s^{\text{T}}LQL^{\text{T}}s$, and let $c=s^{\text{T}}Es$, then κ=a/(a+c) and ω=b/(b+c). We have shown that a≥b, a>0, b>0 and c>0. Therefore, we apply the property of fractions previously shown and conclude that κ≥ω.
